# Modeling on number of children ever born and its determinants among married women of reproductive age in Ethiopia: A Poisson regression analysis

**DOI:** 10.1016/j.heliyon.2023.e13948

**Published:** 2023-02-23

**Authors:** Niguss Cherie, Lemma Getacher, Alemayehu Belay, Teklemariam Gultie, Aleme Mekuria, Samrawit Sileshi, Getu Degu

**Affiliations:** a School of Public Health, College of Medicine and Health Sciences, Wollo University, Dessie, Ethiopia; b School of Public Health, Asrat Woldeyes Health Science Campus, Debre Berhan University, Debre Berhan, Ethiopia; c Department of Public Health, Ethiopian Public Health Institute (EPHI), Addis Ababa, Ethiopia; d School of Nursing and Midwifery, College of Medicine and Health Sciences, Arba Minch University, Arba Minch, Ethiopia; e School of Public Health, College of Health Sciences, Mizan Tepi University, Mizan Teferi, Ethiopia; f School of Public Health, College of Medicine and Health Sciences, Bahir Dar University, Bahir Dar, Ethiopia; g Faculty of Public Health**,** Institute of Health, Jimma University, Jimma, Ethiopia

**Keywords:** Poisson regression, Children ever born, Determinants, Ethiopia

## Abstract

**Background:**

One of the main components of population dynamics that determine the size, structure, and composition of a country’s population is the number of ever-born children. Psychological, economic, social, and demographic factors all have a strong influence on and predict it. However, there is little information on its current status in Ethiopia. As a result, modeling the number of children ever born and its determinants is critical for the Ethiopian government to develop appropriate policies and programs.

**Methods:**

A total of 3260 eligible women were used as a study sample in this study to assess the number of children ever born and determinants among married reproductive age women in Ethiopia. Secondary data were culled from the 2019 Ethiopian Demography and Health Survey datasets. The factors associated with the number of children born were identified using a Poisson regression model (CEB).

**Results:**

The average number of children per mother was 6.09, with a standard deviation of 8.74. There were 2432 (74.6%) rural residents among the total respondents, 2402 (73.7%) have no formal education, and three out of five women are not currently working. The participants' average age was 41.66, with a standard deviation of 3.88. When compared to urban residents, the number of CEB for rural residents is 1.37 times higher. When compared to women with no education, the number of CEB for women with higher education was reduced by 48%. For every unit increase in respondents' current age, the percent change in the number of children ever born increases by 2.4%. For every unit increase in the family's wealth index status, the percent change in the number of children ever born decreases by 1.7%.

**Conclusion:**

When compared to the target of Ethiopia’s health transformation plan, the average number of children born is higher. Improving the household wealth index, women’s education, and employment status all contribute to a reduction in the number of CEB, which is important in balancing population growth with natural capacity and the country’s economic development.

## 1 Introduction

The number of ever-born children (CEB) is a key component of population dynamics that determines a country’s population size, structure, and composition. Children ever born are defined as children born to women during a specific age bracket and are the average number of children born alive to women during that age bracket. Similarly, it is a count of the number of children born alive among married women of reproductive age. It includes all live births, living or dead, from married women up to the time of data collection [1].

The increased number of CEB has caused the world’s population to grow at a rapid rate, with the population of Sub-Sahara Africa (SSA) more than doubling in the next forty years when compared to the current population [2,3]. Total fertility is now 2.5 children per woman globally, according to the 2015 revision of world population prospects. With a fertility rate of 4.7 children per woman, Africa continues to have a high number of CEB. There is widespread concern in SSA about fertility levels, patterns, and trends [3]. This pattern can be influenced by both proximal and distal factors of the number of CEB, which is the most important determinant of population dynamics in one country [4].

Several demographic and economic factors influence the number of children born each year. One of these factors was discovered to be women’s educational status. Improving women’s educational status causes them to want fewer children because the opportunity cost for higher education is adjusted by reducing the opportunity for a larger number of children. Furthermore, when labor demand is high and supply is low, the number of CEB is relatively higher among young couples, implying that the timing of the number of CEB is closely related to economic conditions [5]. Mother's education has a negative impact on the average number of children ever born to a woman; women with married status have the highest number of children [6].

According to an Ugandan study, the number of children decreases as respondents' and their husbands' education levels rise. The study discovered that increased education and delayed marriage among women contributed significantly to the decrease in the number of children between 2006 and 2011. The number of CEB has decreased in recent decades. Because continued improvement in secondary school completion will result in older age in initial marriage [7]. Another study found that education, employment, and food security were important responsible childbearing factors among women [8]. Another study discovered that a mother’s education has a negative effect on the average number of children born to women in Botswana. Similarly, the birth level was discovered to have very significant effects on children ever born among reproductive women in Semnan, Iran [6,9].

One of the independent predictors is also the place of residence. According to a Botswana study, women living in cities/towns and urban villages had 11.2% and 6.8% fewer children than women living in rural areas, respectively [6]. Age is another factor that influences the number of children born. According to studies conducted in Japan and Korea, younger women have fewer child-bearing intentions, but it is higher among those who live in rural areas with larger family members [10,11]. The percentage of the number of kids was consistently decreasing with a decrease of age groups. Women in the age group 45–49 have a higher number of kids than any other lower age group [1,6].

Non-working mothers have more children than working mothers, and mothers who watch television at least once a week have 9.9% fewer children than those who do not watch television at all. Women with married status have the highest fertility, having 21.7% more children than women who have never married [6]. The number of children born was significantly related to the wealth index and the husband's wish [12,13]. Because of societal modernization, the number of children per woman has decreased significantly around the world, reaching just under 2.5 children per woman [14]. This rate of transition is shocking not only for developed countries, but also for developing countries like Ethiopia.

Several studies conducted in Ethiopia revealed that the fertility rate has risen. According to a study conducted in southern Ethiopia, the prevalence of high fertility was 69.1% [15]. A similar study in central Ethiopia's Addis Ababa found that 72.4% of respondents had a high fertility rate [16]. Age, educational status, rural residence, educational status, employment, marriage, contraceptive use, and postpartum infecundability were the major factors associated with fertility rate, index of marriage, postpartum infecundability was, desire for children, history of under-five child, poor knowledge of contraception, and low wealth tertile [[16], [17], [18], [19]].

None of the preceding studies, however, used a Poisson regression model of on number of children born. Because Ethiopia is experiencing rapid population growth, modeling total children ever born is critical. Because data on the direction of Ethiopia's fertility and other demographic indices is limited, mathematical modeling is being explored to track fertility outcomes. As a result, the purpose of this study is to assess the number of children ever born and associated factors among Ethiopian reproductive-age women.

## 2 Methods and materials

### 2.1 Study settings and study design

Ethiopia is located in the horn of Africa and has a total land area of 1,100,000°km^2^. The country’s latitude and longitude are 33° and 48°E, respectively, and 3° and 15°N. According to the 2007 population census, Ethiopia has a population of 112,078,730 people. There were 26,226, 422 (23.4%) women of reproductive age among them [20]. The country is divided into nine ethnically based and politically autonomous regional states (Afar, Amhara, Benishangul Gumuz, Gambella, Harari, Oromia, Somalia, Southern Nations, Nationalities, and People's Region (SNNP), and Tigray) and two administrative cities, Addis Ababa and Dire Dawa. A cross-sectional study design was used with the 2019 Ethiopian Demography and Health Surveys (EDHS) [21].

### 2.2 Population and eligibility criteria

The study’s source population consisted of reproductive-aged women from all regions of Ethiopia. Furthermore, the study population for this research consisted of reproductive-age women living in the country’s selected enumeration areas. EDHS analysis included all women in the selected households who had been residents of the country for six months or more during the study period. The sample size for this study was 3260, drawn from 2019 EDHS datasets from Ethiopia’s nine geographical regions and two administrative cities. The sample for this study was drawn using a two-stage stratified sampling method. In each of the enumeration areas chosen, a household listing operation was carried out. The EDHS used a multistage stratified cluster sampling technique in which sample households were chosen within clusters (enumeration areas) [21].

### 2.3 Operational definitions

**The total number of children ever born (CEB):** Are children born to women during a specific age classification, and the average number of children born alive to a specific reproductive age group of women in that specific age category. Until the time of data collection, the CEB to a specific woman may be an indicator and measure of her lifetime fertility experience.

### 2.4 Variables and covariates of the study

The dependent variable of the study was the total number of children ever born (CEB). Whereas the independent variables were place of residence, educational level, frequency of listening to the radio, respondents currently working status, current age of respondents, and wealth index.

### 2.5 Data sources and analysis

#### 2.5.1 Data source

This research makes use of data from the 2019 Ethiopian Demographic and Health Surveys (EDHS). The surveys were carried out with nationally representative samples drawn from all regions of the country. The EDHS reports include information on the sample design, including the sampling framework and sample implementation, as well as response rates. This study drew on data from Ethiopia, a population-based, cross-sectional survey funded by the US Agency for International Development that collects nationally representative demographic and health data on women and young children. The EDHS used a multistage stratified cluster sampling technique in which sample households were chosen within clusters (enumeration areas) [21]. Each cluster received a sampling weight.

### 2.6 Data processing and analysis

Data were extracted from the EDHS database and analyzed with IBM statistics SPSS version 25. To begin, descriptive statistics were calculated using frequencies and percentages to summarize the sociodemographic characteristics of study participants. The Poisson regression model was then validated using the following assumptions.

Poison regression is based on five assumptions: These are count data, one or more independent variables (continuous, ordinal, or nominal), variation independence, count distribution follows a Poisson distribution (Pearson chi-square = 1.1, indicating equidistributional), and the model's mean and variance are identical. There is, however, overdispersion (deviance = 1.3). Because there is no overdispersion, the poison regression model is more appropriate for this data than the negative binomial regression model. The omnibus test yielded p < 0.001, indicating a statistically significant overall model.

The association of independent factors to the outcome variable was declared using an incidence rate ratio (IRR) with a 95% confidence interval. Variables with p-values less than 0.05 at the 95% confidence interval were identified as factors influencing the number of children born in Ethiopia among reproductive-age women.

### 2.7 Ethical considerations

There is no ethical committee that has approved this study because it uses secondary data from EDHS.

## 3 Results

### 3.1 Socio-economic and demographic characteristics

We considered about six predictor variables from the EDHS datasets, namely age of respondents in years, respondent's highest educational level, wealth index, place of residence, frequency of listening to radio, and respondent's current working status, which were found to have significant effects on the dependent variable. This study included 160 clusters and an average of 21 households with a number of CEB ranging from 1 to 39. Each cluster received a sampling weight. Of the total respondents, 2432 (74.6%) live in rural areas, 2402 (73.7%) have no formal education, and three out of every five women are unemployed. (Table 1).

**Table 1:** Socio-economic and demographic characteristics of reproductive age married women in Ethiopia (n = 3260).

Variables	Response	Frequency (%)
Place of residence	Rural Urban	2432 (74.6) 828 (25.4)
Highest educational level	Higher Secondary Primary No education	109 (3.3) 147 (4.5) 602 (18.5) 2402 (73.7)
Frequency of listening to the radio	At least once a week less than once a week Not at all	570 (17.5) 918 (28.2) 1772 (54.4)
Wealth index	Richest Richer Middle Poorer Poorest	858 (26.3) 501 (15.4) 503 (15.4) 550 (16.9) 848 (26.0)
Respondent currently working	Yes No	1268 (38.9) 1992 (61.1)
Age of respondents in years	45–49 40–44 35–39	1029 (31.6) 1185 (36.3) 1046 (32.1)

### 3.2 Number of children ever born

A total of 3260 women took part in this study. The participants' average age was 41.66, with a standard deviation of 3.88. The dependent variable was the number of children ever born to Ethiopian women. This variable had 17 categories in the main dataset (0–16). The data recorded the number of children born to women aged 35–49 who had contributed to the population by giving birth, with no child being the worst possible outcome. With seven children born, the highest percentage of CEB was 14.0%.

The mean number of children per mother was 6.09, with a variance of 8.74, which is higher than the mean value and indicates a slight but not significant overdispersion. Therefore, this is not obliged us to use a Negative Binomial regression model (Table 2).

**Table 2:** Cross-tabulation of predictor variables with children ever born among reproductive age married women in Ethiopia (n = 3260).

Predictors		Number of children ever born										
		0	1	2	3	4	5	6	7	8	9	9^+^
		N (%)	N (%)	N (%)	N (%)	N (%)	N (%)	N (%)	N (%)	N (%)	N (%)	N (%)
Place of residence	Urban	60 (57.1)	97 (68.8)	138(63.0)	120(58.8)	116(39.5)	80(23.7)	63(15.1)	53(11.6)	43(11.1)	20(6.9)	38(9.4)
	Rural	45 (42.9)	44 (31.2)	81(37.0)	84(41.2)	178(60.5)	258(76.3)	353(84.9)	405(88.4)	346(88.9)	271(93.1)	367(90.6)
Highest educational level	No education	51 (48.6)	56 (39.7)	92(42.0)	97(47.5)	194(66.0)	248(73.4)	340(81.7)	381(83.2)	337(86.6)	247(84.9)	359(88.6)
	Primary	28 (26.7)	42(29.8)	47(21.5)	51(25.0)	71(24.1)	77(22.8)	73(17.5)	75(16.4)	51(13.1)	44(15.1)	43(10.6)
	Secondary	15 (14.3)	30(21.3)	41(18.7)	31(15.2)	18(6.1)	6(1.8)	1(0.2)	2(0.4)	0	0	3(0.7)
	Higher	11 (10.5)	13(9.2)	39(17.8)	25(12.3)	11(3.7)	7(2.1)	2(0.5)	0	1(0.3)	0	0
Frequency of listening to the radio	Not at all	41(39.0)	57(40.4)	82(37.4)	93(45.6)	145(49.3)	205(60.7)	233(56.0)	277(60.5)	228(58.6)	173(59.5)	238(58.8)
	Less than once a week	42(40.0)	38(27.0)	74(33.8)	60(29.4)	89(30.3)	74(21.9)	112(26.9)	124(27.1)	110(28.3)	88(30.2)	107(26.4)
	At least once a week	22(21.0)	46(32.6)	63(28.8)	51(25.0)	60(20.4)	59(17.5)	71(17.1)	57(12.4)	51(13.1)	30(10.3)	60(14.8)
Wealth index	Poorest	7(6.7)	15(10.6)	32(14.6)	27(13.2)	59(20.1)	103(30.5)	117(28.1)	142(31.0)	117(30.1)	98(33.7)	131(32.3)
	Poorer	13(12.4)	7(5.0)	20(9.1)	23(11.3)	40(13.6)	57(16.9)	80(19.2)	88(19.2)	86(22.1)	62(21.3)	74(18.3)
	Middle	9(8.6)	12(8.5)	15(6.8)	15(7.4)	38(12.9)	52(15.4)	80(19.2)	83(18.1)	71(18.3)	46(15.8)	82(20.2)
	Richer	10(9.5)	13(9.2)	13(5.9)	19(9.3)	29(9.9)	51(15.1)	72(17.3)	87(19.0)	71(18.3)	63(21.6)	73(18.0)
	Richest	66(62.9)	94(66.7)	139(63.5)	120(58.8)	128(43.5)	75(22.2)	67(16.1)	58(12.7)	44(11.3)	22(7.6)	45(11.1)
currently working	No	39(37.1)	56(39.7)	100(45.7)	96(47.1)	173(58.8)	213(63.0)	269(64.7)	302(65.9)	264(67.9)	209(71.8)	271(66.9)
	Yes	66(62.9)	85(60.3)	119(54.3)	108(52.9)	121(41.2)	125(37.0)	147(35.3)	156(34.1)	125(32.1)	82(28.2)	134(33.1)
Age in years	45–49	42(40.0)	61(43.3)	84(38.4)	94(46.1)	115(39.1)	127(37.6)	160(38.5)	156(34.1)	109(28.0)	49(16.8)	49(12.1)
	40–44	30(28.6)	47(33.3)	82(37.4)	64(31.4)	110(37.4)	125(37.0)	138(33.2)	189(41.3)	142(36.5)	112(38.5)	146(36.0)
	35–39	33(31.4)	33(23.4)	53(24.2)	46(22.5)	69(23.5)	86(25.4)	118(28.4)	113(24.7)	138(35.5)	130(44.7)	210(51.9)
		Mean = 6.09, Variance = 8.74, SD = 2.96										

Rural women (405 (88.4%)), women with no education (359 (88.6%)), and women with no history of radio listening (238 (58.8%)) have the highest CEB (Table 2).

### 3.3 Factors associated with several children ever born

The Poisson regression model assumes that the Poisson mean has a gamma distribution with variation across subjects. The value of 1.3 in this study indicates that there is no significant overdispersion. As a result, we can analyze the data using a Poisson regression model rather than a Negative Binomial regression model (Table 3).

**Table 3:** A Poisson regression model analysis of factors associated with Children Ever Born (CEB) among reproductive age married women (35–49 years) in Ethiopia (n = 3260).

Predictors		B	IRR = Exp (B)	95% Wald Confidence Interval for Exp (B)	
				Lower	Upper
Place of Residence	Rural	0.313	1.367*	1.291	1.448
	Urban	Reference			
Highest Educational level	Higher	−0.651	0.520**	0.459	0.589
	Secondary	−0.652	0.521**	0.456	0.595
	Primary	−0.102	0.903**	0.863	0.945
	No education	reference			
Frequency of listening to the radio	At least once a week	0.035	1.035	1.987	1.086
	less than once a week	0.001	0.999	0.963	1.036
	Not at all	Reference			
Respondents currently working	Yes	−0.048	0.954*	0.923	0.986
	No	reference			
Respondents current age	Respondent's current age	0.024	1.024**	1.020	1.028
Wealth index	Wealth index	−0.015	0.983*	0.970	0.995

*P-value ≤0.005, **P-value <0.001, IRR = incidence rate ratio, CI = confidence interval.

When compared to urban residents, the number of CEB for rural residents is 1.367 times (IRR = 1.367, 95% CI: 1.3, 1.448). When all other variables were held constant, the number of CEB for women with higher education, secondary education, and primary education was 0.520 times (IRR = 0.520, 95%CI: 0.459, 0.589), 0.521 times (IRR = 0.521, 95%CI: 0.456, 0.595), and 0.903 times (IRR = 0.903, 95%CI: 0.863, 0.945) (Table 3).

Similarly, the number of CEB for women who are currently working is 0.954 times (IRR = 0.954, 95% CI: 0.923, 0.986) higher than the number of CEB for women who are not currently working. The percent change in the number of children ever born, on the other hand, increases by 2.4% for every unit increase in respondents' current age. For every unit increase in the family's wealth index status, the percent change in the number of children ever born decreases by 1.7% (Table 3).

## 4 Discussion

Children ever born (CEB) is one of the three main determinants of population dynamics that determine the size, structure, and composition of the population in any country. It can be influenced by a variety of factors. These variables are classified as sociodemographic, socioeconomic, fertility preference, and family planning-related. The educational level, place of residence, respondents' working status, current age, and wealth index were all addressed in this study. According to the findings of this study, the highest educational level, place of residence, respondents' working status, current age, and wealth index all have a significant effect on CEB in Ethiopia.

When compared to urban dwellers, being a rural dweller increases the number of children ever born. The number of children born for rural residents is 36.7% higher (IRR = 1.367) than from urban residents. According to a study conducted in Bangladesh, the number of CEB for rural residence increased by 1% [1]. According to Ethiopian fertility research, living in the rural area increases the number of children ever born by 4.9% compared to women living in the city [22]. This finding is supported by studies conducted in Kenya, which found that living in rural areas increases the number of CEB by 10% [23]. This finding is supported by a Botswana study, which found that the number of CEB in urban residents decreased by 6.8% [6]. This could be because rural women have less access to contraception and education opportunities, which influences an individual’s behavior, including family planning utilization [24]. Furthermore, the number of CEBs awarded to women in Bangladesh does not differ significantly between urban and rural areas [1]. The reason could be that families in urban areas have fewer resources, such as a house, which forces them to limit the number of children [25].

The number of CEB is affected by women's educational level. Women with a higher education, a secondary education, and a primary education reduce the number of children born by 48%, 47.9%, and 9.7%, respectively. This finding is consistent with previous research conducted in Kenya and Egypt. A study conducted in Kenya found that women who completed primary, secondary, and higher-level education reduced the number of children born by 26%, 25%, and 40%, respectively. Another study conducted in Egypt revealed that higher or secondary educated women have two fewer children than less educated women, implying that as educational status increases, the number of children born decreases. All of the above studies concluded that education had a significant lowering effect on the number of CEB [23,26]. This means that the number of children per woman decreases as education level increases. Women became more educated as they gained access to prenatal care, child health services, and all other necessary health information. As a result, fewer children may have been born. Female education has a greater impact on delaying marriage age, which results in delayed fertility [27].

The number of CEB increased as the respondent's age increased. For every unit increase in respondents' current age, the percent change in the incident rate of children ever born increases by 2.4%. This finding is similar to a study conducted in Nepalese women, which found that the incident rate of ever-born children increases by 4.9% for every year increase in women's current age [28]. This means that as a woman's age increases by one year, the number of CEB nearly doubles in Nepal and doubles in Ethiopia among married reproductive-age women.

Similarly, in this study, the wealth index is influenced by the number of children born. For every unit increase in the family's wealth index status, the percent change in the incident rate of children ever born decreases by 1.7%. The rising wealth index has a negative impact on the number of children born in Ethiopia. This finding is supported by a study conducted in Kenya, which found that being in a middle or rich wealth index family reduces the number of children born by 7% and 17%, respectively, when compared to being in a poor wealth index family [23]. This could be because when a household has a high wealth index, the likelihood of their understanding of family planning increases, resulting in a decrease in the number of children born. Furthermore, those in the high wealth index may be overworked as a result of their business. This may result in time constraints when having a child. They may have fewer children as a result of this. Wealthy families postpone fertility in order to complete their education. Poverty is widespread, resulting in high infant mortality, while high fertility maximizes replacement of offspring who die before reproducing [24].

In terms of the respondent’s current working status, the number of CEB for women who are currently working is 4.6% (0.95) lower than the number of CEB for women who are currently not working. Evidence from Botswana found that working reduces the number of children born by 8% (0.92) compared to those who do not work. Another finding from research conducted in Kenya reported that when women have no work experience, the number of children increases when compared to women who have work. This indicated that the number of CEB had decreased among women who were currently working [23]. This could be due to the fact that working women may have less time to raise their children than those who are not currently working. This suggests that the transition to motherhood is strongly discouraged by employment [29].

Regarding the limitations of this study, the findings have some limitations. Children born to unmarried women, including divorced, separated, and widowed women, were not included in this data analysis because only ever-married women were interviewed in the EDHS survey. As a result of the omission of these births, the number of CEB from women in the country may be slightly underestimated.

## 5 Conclusion

When compared to the target of Ethiopia’s health transformation plans, the average number of children born is higher. This study also examines the impact of the highest educational level, place of residence, respondents' employment status, current age, and wealth index on the number of CEB in Ethiopia using data from the 2019 national EDHS. Accordingly, respondents' place of residence and current age had a positive impact on the number of CEB, whereas educational level, respondents' working status, and wealth index had a negative impact on the number of CEB. Improving the wealth index and putting more women to work contribute to a reduction in the number of CEB, which is important in balancing population growth with natural resource capacity and the country's economic development.

Based on the findings, it is recommended that all stakeholders work together to ensure the proper implementation of Ethiopia's national population policy, which aims to reduce the total number of children born per woman to 4.0 by 2015. It is critical to work on promoting and supporting female students' participation in higher education. Women, on the other hand, should have more opportunities for employment. Researchers should use mixed methods research approaches to investigate other cultural and religious factors that influence fertility and family planning services.

## Author contribution statement

Niguss Cherie, Lemma Getacher, Teklemariam Gultie, Alemayehu Belay: Conceived and designed the experiments; Performed the experiments; Analyzed and interpreted the data; Wrote the paper.

Aleme Mekuria, Samrawit Sileshi, Getu Degu: Contributed reagents, materials, analysis tools or data; Wrote the paper.

## Funding statement

This research received no specific grant from any funding agency in the public, commercial, or not-for profit sectors.

## Data availability statement

Data will be made available on request.

### Declaration of interest’s statement

The authors declare no conflict of interest.

## Acronyms/Abbreviations

CEB: Children Ever Born
EDHS: Ethiopia Demographic and health survey
